# Reduced air contamination in an ICU environment with a portable air purification system

**DOI:** 10.1186/cc10686

**Published:** 2012-03-20

**Authors:** J Papaparaskevas, V Papas, M Pratikaki, A Tsakris, C Routsi

**Affiliations:** 1 Medical School, University of Athens, Greece; 2 Evangelismos Hospital, Athens, Greece

## Introduction

Indoor air contamination has been implicated in hospital-acquiring infections, especially in immunocompromised patients. This implies that, along with other preventing measures, maintenance of good air quality in critical areas in hospitals is helpful to reduce the incidence of these infections. The objectives of this study were to evaluate the quality of an ICU air environment regarding total and fungal flora and the ability of a mobile air purification system (Hegoa; ANEMO, Oullins, France). This device uses UVc technology (photocatalysis) to destroy a wide range of microorganisms, including fungi.

## Methods

Air samples were obtained before and after the Hegoa air purification system was started in seven ICU rooms, including a total of 10 beds, during a 24-hour period and at 3-hour intervals. From each room and time point, 200 l air samples were collected using a calibrated biocollector (Air Ideal; bioMerieux, Marcy L'Etoile, France). Cultures were performed on Triptycase Soy Agar and Sabouraud chloramphenicol agar plates, for the total and the fungal flora, respectively. Plates were incubated at 36°C and room temperature for a period of 7 days.

## Results

A total of 112 air samples from sampling sites in the ICU rooms were collected during the 24-hour study period. Before starting the air purification unit, total flora ranged from 175 to 70 cfu/m^3 ^and fungal flora from 30 to 35 cfu/m^3^. Total flora values were continuously decreasing and at 24 hours after air purification onset were significantly reduced to 30 to 50 cfu/m^3 ^(72% reduction). Similarly, environment fungal levels were continuously decreased and at 24 hours after the start were undetectable.

## Conclusion

The Hegoa mobile air purification system shown a rapid lowering of contaminates with eventual elimination of fungal flora. Therefore, that equipment may provide an efficient method of reducing air contamination into the ICU. Whether equipping ICU rooms with such devices could protect immunocompromised patients admitted to the ICU against fungal and microbial risk has to be examined.

